# In vitro evaluation of the microhardness of conventional and bulk-fill resins based on the depth of light-curing and the curing unit

**DOI:** 10.4317/jced.64256

**Published:** 2026-07-29

**Authors:** Vanessa Verónica Sara García-Mendoza, Rosa Josefina Roncal-Espinoza

**Affiliations:** 1 Universidad Católica Santo Toribio de Mogrovejo; 2 Universidad Privada Norbert Wiener

## Abstract

**Background:**

To evaluate the microhardness of conventional and bulk-fill composite resins based on the depth of light-curing (2 mm and 4 mm) and the curing unit used (VALO Grand and BluePhase N G4 lamp).

**Material and Methods:**

Discs were fabricated using two bulk-fill resins: Beautifil Bulk (BB) and Filtek Bulk Fill (ZB); and two conventional resins: Beautiful II (BC) and Filtek Z350 XT (ZC). For the preparation, the light-curing depth was set at 2 and 4 mm, and the curing units used were VALO Grand and BluePhase N G4. Microhardness was measured using the Vickers test on light-cured samples under standardized conditions. Statistical analysis was performed using ANOVA and the Tukey test (p < 0.05).

**Results:**

Significant differences were found between the groups, with the BB 4mm Valo group exhibiting the lowest microhardness values and significant differences compared to the others. Meanwhile, the 4 mm Bluephase ZC resin exhibited the highest microhardness values compared to the bulk-fill resin. At a thickness of 2 mm, the conventional resins showed similar values regardless of the polymerization source, except for BB, which performed better with the Valo unit. In addition, Valo demonstrated good performance with ZC at both thicknesses and results comparable to Beautifil II at 2 mm. Bluephase showed consistent values across all groups and depths, with superior performance when applied over ZC.

**Conclusions:**

The results showed that the final microhardness of the restorative material is influenced by a combination of resin type, depth, and light-curing unit; the conventional composite resin that exhibited the highest microhardness values was ZC, light-cured using a Bluephase light source at a thickness of 4 mm, compared to the bulk-fill resin.

## Introduction

Composite resins are widely used in dentistry and can be employed for the restoration of both anterior and posterior teeth. This material consists of an organic matrix, an inorganic filler, and a bonding agent, components that have evolved to improve its properties (1 - 3). Over the years, modifications have been made to the matrix, such as a reduction in filler particles, changes in shape or surface treatment, alterations in the chemical structure of the monomers, and changes in the dynamics of the polymerization reaction (4). Although the placement techniques and performance of composite resins are becoming increasingly widespread, their clinical performance and longevity are still questioned (5). Composite resins can be light-cured to different depths. Depending on the power of the light-curing source, light energy gradually decreases as it passes through the composite resin (6), which is why the incremental or 2 mm layer technique was implemented (7). Although this technique ensures a proper fit for each increment, preventing microleakage, recurrent caries, and postoperative sensitivity (8), it requires more clinical time and careful attention to avoid the formation of air bubbles. In this regard, as a strategy to counteract these effects, modifications have been made to the filler particles, matrix, and initiators of composite resins by incorporating materials such as bulk-fill resins (9). Bulk-fill resin is a restorative material with greater translucency, larger particle size, a lower percentage of filler particles, and additional initiators (5 , 6 , 10). During light-curing, this results in greater light penetration when large increments are applied, as well as a reduction in polymerization shrinkage stress, bubble formation, and the risk of contamination (11). However, some studies (9 , 12) demonstrate limitations such as a decrease in mechanical properties as the material thickness increases (12), difficulties in light penetration, and a reduced degree of conversion, which depends not only on the resin's composition but also on factors related to photoactivation, including the light-curing unit, the type of photoactivation selected, and the amount of energy applied (13). One of the mechanical properties evaluated in composite resins that could have the greatest impact on the success of the restoration is microhardness, which, according to previous studies (12 , 13), decreases as the resin thickness increases. Therefore, research on light-curing units could be an important factor in microhardness results, as seen in Shimokawa's study (13), which demonstrated that the Valo Grand light source produced more homogeneous microhardness values in bulk-fill resin samples. Meanwhile, a study (14) evaluating the effect of different polymerization protocols on the microhardness of composite resins concluded that these values are more closely related to the material composition than to the light-curing protocol. Therefore, the objective of this study was to evaluate the microhardness of conventional and bulk-fill resins according to the depth of light-curing and the LED light-curing unit.

## Material and Methods

**Figure 1:**
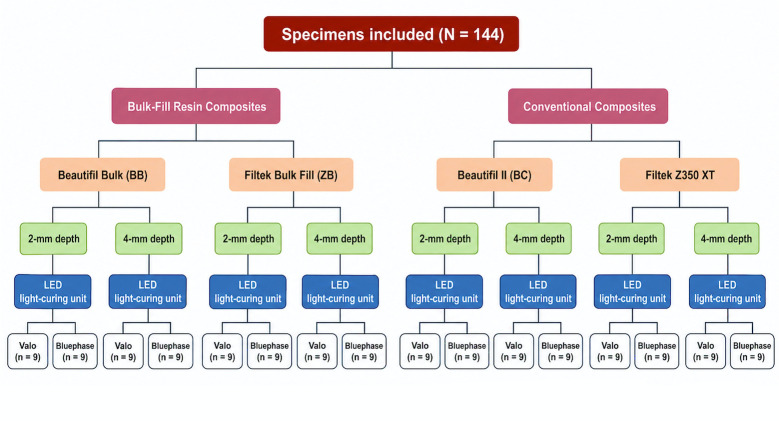
Experimental grouping of the 144 resin composite specimens according to material category (bulk-fill or conventional), composite brand, restoration depth (2 or 4 mm), and LED light-curing unit (Valo or Bluephase). Each experimental subgroup consisted of nine specimens.

The study was approved by the Research Ethics Committee of the School of Medicine at the Santo Toribio de Mogrovejo Catholic University in Peru, under Resolution No. 295-2024-USAT-FMED. This in vitro experimental study used a sample size calculated using the G* Power 3.1 software (Heinrich-Heine-Universität Düsseldorf, Germany), with 80% power and a 5% Type I error rate, and an effect size from a pilot study, resulting in 9 samples per group, for a total of 144 samples (n=9). - Specimen preparation Specimens were fabricated using two bulk-fill resins: Beautifil-Bulk Restorative (Shofu, Japan) and Filtek Bulk Fill (3M, USA), and two conventional resins: Beautifil II (Shofu, Japan) and Filtek Z350 XT (3M, USA). In addition, for the preparation of the samples, light-curing depths of 2 and 4 mm were considered, and the following curing units were used: VALO X LED Light-Curing Unit (Ultradent Products, Inc., South Jordan, UT, USA) and BluePhase N G4 Light (Ivoclar Vivadent AG, Schaan, Liechtenstein) (Fig. 1).

1

The discs were fabricated using two metal dies with a central hole 5 mm in diameter, one 2 mm thick and the other 4 mm thick. The mold was placed on a glass slab, and the composite resin was packed into the mold in a single increment for the bulk-fill resins and in 2-mm increments for the conventional resins, using a resin spatula (Osung MND-Korea). A translucent celluloid tape was placed on top, followed by a glass slab used on the open end of the mold, and a 5 Kg weight was used to apply pressure from the top to achieve a smooth surface free of excess material. Light curing was performed using different types of LED curing units: VALO X (Ultradent Products, Inc., South Jordan, UT, USA) in standard power mode (1000 mW/cm²) and the BluePhase N G4 lamp (Ivoclar Vivadent AG, Schaan, Liechtenstein) in soft mode (1200 mW/cm²). Light curing was performed by positioning the tip of the light source for 20 seconds. - Surface Microhardness measurement Once the discs were finished, they were stored in a dry, airtight environment to prevent contamination. Microhardness testing was performed 24 hours after fabrication. A force of 0.1 kgf was applied for 30 seconds at eight points on each disc, four on the top surface and four on the bottom, using a Vickers microhardness tester. - Statistical Analysis All data obtained were stored in Microsoft Excel and subsequently analyzed using the statistical software SPSS 27.0. The results were presented using descriptive statistics; the Shapiro-Wilk test was then performed to assess the normality of the data. Since the data were normally distributed, they were subjected to analysis of variance (ANOVA), the Tukey test, and the t-test for multiple comparisons ( = 0.05).

## Results

**Figure 2:**
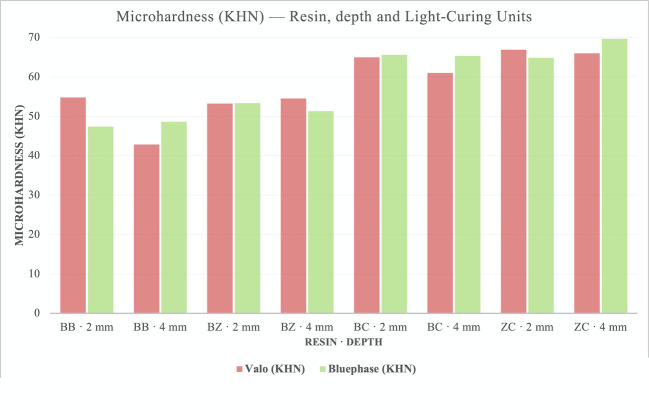
Microhardness evaluation of conventional and bulk-fill resins based on the depth of light-curing and the LED curing unit.

Figure 2 shows the values of microhardness (KHN) for each of the groups evaluated, including conventional resins and bulk-fill resins.

2

It can be seen that the group with the highest microhardness was ZC 4mm Bluephase. The lowest value was found in BB 4mm Valo. Furthermore, most of the experimental groups evaluated had microhardness values above 60. Table 1 shows a comparison of the microhardness of conventional and bulk-fill resins at a light-curing depth of 2 mm.

Table 1

The BB resin showed statistically significant differences between light curing with Valo and Bluephase, with higher values obtained using Valo. Furthermore, it was determined that the highest microhardness value was obtained by the ZC resin using the Valo light curing unit, with no statistically significant differences compared to Bluephase, and with BC using both light curing units. Table 2 shows the microhardness values at a depth of 4 mm for the different resins and curing light sources.

Table 2

Statistically significant differences were observed in BC when comparing the polymerization sources, with Bluephase exhibiting higher values. When evaluating ZB, there were no differences among the polymerization sources. Meanwhile, for ZC, the highest values were reported with Bluephase. Overall, the highest values were found with ZC Bluephase, while the lowest values were determined with BB Valo. In Table 3, when comparing microhardness measurements using the Valo light-curing unit, statistically significant differences were found when evaluating BB 2 mm and 4 mm, with the highest values observed for 2 mm.

Table 3

No differences were found in the ZB and ZC groups. However, the highest values were in ZC at 2 mm, while the lowest values were reported in BC at 4 mm. Table 4 shows the microhardness values obtained using the Bluephase curing light. When evaluating BB at 2 mm and 4 mm, there are no statistically significant differences, and the same is true for BC and ZB; however, for ZC, there are differences when comparing 2 mm to 4 mm, with the highest values observed for ZC at 4 mm and the lowest values for BB at 2 mm.

Table 4

## Discussion

This study aimed to evaluate the microhardness of conventional and mass-filled composite resins, taking into account the depth of light-curing (2 mm and 4 mm) and the light-curing unit used (Valo and Bluephase). The results revealed statistically significant differences between the evaluated groups, confirming that both the depth and the composition of the material, as well as the light source, directly influence its mechanical behavior. It was observed that the 3M Z350 XT resin, a nano-hybrid type, exhibited the highest microhardness values, especially when light-cured with Bluephase to a depth of 4 mm. This superiority can be attributed to its composition based on silica and zirconia nanoclusters, which allow for a more compact, dense, and resistant structure, as well as greater efficiency in light transmission, facilitating the conversion of monomers in deep layers. This formulation has demonstrated an excellent response to deep light-curing, consistent with the findings of Elhejazi et al. (15) and Jakupovi et al. (14). In contrast, Beautifil Bulk Shofu, despite having a high filler content (~87%), exhibited the lowest microhardness values, particularly at 4 mm when cured with the Valo lamp. This could be explained by the use of bioactive S-PRG (pre-reacted glass-ionomer) fillers, which, while offering beneficial properties such as fluoride release and an anti-plaque effect, have lower hardness compared to ceramic particles such as zirconia and silica. Furthermore, these fillers may scatter light less efficiently, limiting monomer conversion in depth (16). When considering the main monomers, it is observed that resins containing Bis-GMA and UDMA, such as 3M Z350 XT and Beautifil II Shofu, consistently exhibited higher microhardness. These monomers have more rigid molecular structures, promoting greater cross-linking and surface strength. In contrast, 3M Bulk Fill resin contains modified monomers such as AUDMA and AFM, designed to reduce polymerization shrinkage, but which could result in a less dense matrix, which would explain its intermediate microhardness values (17). About light-curing units, Bluephase demonstrated greater effectiveness than Valo in most groups, particularly at depths of 4 mm. These differences may be due to factors such as the composition of the resins and the filler content of bulk-fill and conventional resins. It should also be noted that its Polywave technology emits a broader spectrum and better activates different types of photoinitiators, such as camphorquinone, which is present in all the resins evaluated (14 , 18). It should be noted that some combinations, such as Beautifil II Shofu, showed no significant differences between the two curing sources, which could indicate greater material stability in the face of variations in the light-curing protocol. In contrast, significant differences were observed with Beautifil Bulk Shofu, suggesting that this material is more sensitive to the type of light source used. Although microhardness is a relevant parameter for estimating the effectiveness of light-curing and surface strength, it must be considered alongside other clinical factors such as wear resistance, marginal adaptation, and polymerization shrinkage. In line with the proposals by Alzahrani et al. (19) and Berto-Inga et al. (20), it is recommended that future research include additional tests to evaluate the comprehensive performance of resins under real clinical conditions; furthermore, no studies similar to this one were found where we could compare results or evaluate other types of resins for study. Finally, the main limitation of this study is it's in vitro experimental design; although the study was conducted in a laboratory under controlled conditions, it did not simulate the clinical aspects observed in the oral cavity, which play an important role in obtaining results that closely resemble those seen in clinical practice; however, all recommended protocols were followed to ensure that the results are as close as possible to what occurs under real-world conditions.

## Conclusions

Based on an analysis of the results obtained, it is concluded that the final microhardness of the restorative material depends on a combination of factors, such as the type of resin, the depth, and the light-curing unit. Among conventional composite resins, the one that exhibited the highest microhardness values was the 3M Z350 XT resin light-cured with a Bluephase light source, at a thickness of 4 mm, compared to the Bulk Fill resin. Regarding depth, at a thickness of 2 mm, conventional resins exhibit similar microhardness values. Finally, the Bulk Fill resins at a depth of 4 mm exhibited similar microhardness values, with the exception of Beautifil Bulk, which was light-cured with Valo and showed the lowest values.

## Figures and Tables

**Table 1:** Comparison of the microhardness of conventional and bulk-fill resins, based on a light-curing depth of 2 mm.

Depth	Resins	Light-Curing Units	Mean	SD	p-value
2 mm	BB	Valo	54.78 a	1.60	0,00
		Bluephase	47.40 b	1.44	
	BC	Valo	64.99 c	1.0	
		Bluephase	65.60 c	2.07	
	ZB	Valo	53.24 a	1.02	
		Bluephase	53.36 a	1.16	
	ZC	Valo	66.88 c	2.63	
		Bluephase	64.86 c	1.56	

Different superscript letters indicate a statistically significant difference between groups; ANOVA and Tukey’s test (p < 0.05).

**Table 2:** Comparison of the microhardness of conventional and bulk-fill resins, based on a light-curing depth of 4 mm.

Depth	Resins	Light-Curing Units	Mean	SD	p-value
4 mm	BB	Valo	42.87 a	3.49	0,00
		Bluephase	48.61 b	5.03	
	BC	Valo	61.00 c	1.03	
		Bluephase	65.33 d	1.87	
	ZB	Valo	54.51 e	2.12	
		Bluephase	51.33 be	1.37	
	ZC	Valo	65.97 d	1.01	
		Bluephase	69.69 f	0.97	

Different superscript letters indicate a statistically significant difference between groups; ANOVA and Tukey’s test (p < 0.05).

**Table 3:** Microhardness of conventional and bulk-fill resins, as measured using the Valo light-curing unit.

Light-Curing Units	Resins	Depth	Mean	DS	p-value
Valo	BB	2 mm	54.78 a	1.60	0,00
		4 mm	42.87 b	3.49	
	BC	2 mm	64.99 c	1.0	
		4 mm	61.00 d	1.03	
	ZB	2 mm	53.24 a	1.02	
		4 mm	54.51a	2.12	
	ZC	2 mm	66.88 c	2.63	
		4 mm	65.97 c	1.01	

Different superscript letters indicate statistically a significant difference between groups; ANOVA and Tukey’s test (p < 0.05).

**Table 4:** Microhardness of conventional and bulk-fill resins, as measured using the Bluephase light-curing unit.

Light-Curing Units	Resins	Depth	Mean	DS	p-value
Bluephase	BB	2 mm	47.40 a	1.44	0,00
		4 mm	48.61ab	5.03	
	BC	2 mm	65.60 c	2.07	
		4 mm	65.33 c	1.87	
	ZB	2 mm	53.36 d	1.16	
		4 mm	51.33 bd	1.37	
	ZC	2 mm	64.86 c	1.56	
		4 mm	69.69 e	0.97	

Different superscript letters indicate a statistically significant difference between groups; ANOVA and Tukey’s test (p < 0.05).

## Data Availability

Declared none.
